# Effects of four different antihypertensive drugs on plasma metabolomic profiles in patients with essential hypertension

**DOI:** 10.1371/journal.pone.0187729

**Published:** 2017-11-09

**Authors:** Timo P. Hiltunen, Jenni M. Rimpelä, Robert P. Mohney, Steven M. Stirdivant, Kimmo K. Kontula

**Affiliations:** 1 Department of Medicine, University of Helsinki, Helsinki, Finland; 2 Helsinki University Hospital, Helsinki, Finland; 3 Metabolon Inc., Durham, NC, United States of America; Shanghai Institute of Hypertension, CHINA

## Abstract

**Objective:**

In order to search for metabolic biomarkers of antihypertensive drug responsiveness, we measured >600 biochemicals in plasma samples of subjects participating in the GENRES Study. Hypertensive men received in a double-blind rotational fashion amlodipine, bisoprolol, hydrochlorothiazide and losartan, each as a monotherapy for one month, with intervening one-month placebo cycles.

**Methods:**

Metabolomic analysis was carried out using ultra high performance liquid chromatography-tandem mass spectrometry. Full metabolomic signatures (the drug cycles and the mean of the 3 placebo cycles) became available in 38 to 42 patients for each drug. Blood pressure was monitored by 24-h recordings.

**Results:**

Amlodipine (P values down to 0.002), bisoprolol (P values down to 2 x 10^−5^) and losartan (P values down to 2 x 10^−4^) consistently decreased the circulating levels of long-chain acylcarnitines. Bisoprolol tended to decrease (P values down to 0.002) the levels of several medium- and long-chain fatty acids. Hydrochlorothiazide administration was associated with an increase of plasma uric acid level (P = 5 x 10^-4^) and urea cycle metabolites. Decreases of both systolic (P = 0.06) and diastolic (P = 0.04) blood pressure after amlodipine administration tended to associate with a decrease of plasma hexadecanedioate, a dicarboxylic fatty acid recently linked to blood pressure regulation.

**Conclusions:**

Although this systematic metabolomics study failed to identify circulating metabolites convincingly predicting favorable antihypertensive response to four different drug classes, it provided accumulating evidence linking fatty acid metabolism to human hypertension.

## Introduction

In view of the facts that elevated blood pressure has evolved as the leading contributor to global burden of disease [1,2] and unsatisfactory control rates achieved by antihypertensive drug treatment [3,4], there is an urgent need for more effective ways to individualize drug therapies in a patient-specific fashion. While pharmacogenomics approaches may be well suited for personalized treatment of the rare varieties of monogenic hypertension [5,6], studies on pharmacogenomics of essential hypertension have shown much less progress [7,8]. Recent genome-wide association studies on genetic associations of specific antihypertensive drug responses have revealed several potential gene loci of interest [9–16], but the data so far published have lacked consistently replicated findings, and presently there appears to be no clinically useful genetic markers to guide the choice of a given blood pressure-lowering drug.

Metabolomics has been widely used to address a number of questions in human health and disease and has recently been introduced in studies of human hypertension (for review, see [17]). As the first steps toward pharmacometabolomics of hypertension, Wikoff et al. [18], Rotroff et al. [19] and Shahin et al. [20] have identified sets of blood metabolites associated with blood pressure lowering in response to atenolol or hydrochlorothiazide. In addition, using two independent population samples, each amounting appr. 1500 subjects, Menni et al. [21] identified a novel pathway involving hexadecanedioate as an important regulator of human blood pressure. In addition, high serum hexadecanedioate levels were associated with elevated systolic and diastolic blood pressure levels in both European-American and African-American populations [22].

In order to systematically characterize the effects of different classes of antihypertensive drugs on the circulating metabolic profile, we have taken advantage of our GENRES (Genetics of Drug Responsiveness in Essential Hypertension) Study platform [13,23] in which we have studied, in a placebo-controlled, cross-over fashion, the antihypertensive effects of four different drugs (a diuretic, a beta blocker, a calcium channel blocker and an angiotensin receptor antagonist). This rotational study design permits the assessment of the effects of the different drug classes on plasma metabolic profiles in the same individuals, and the use of integrated baseline values derived from three different intervening placebo cycles provides a further means to reduce experimental variation.

## Materials and methods

### Design of the GENRES study: Patients, drug treatments and blood pressure measurements

The design and principal results of the GENRES Study have been published previously [13,23,24]. In brief, a total of 313 hypertensive Finnish men (aged 35 to 60 years) were initially recruited. Inclusion criteria were diastolic blood pressure ≥95 mmHg in repeated measurements or use of antihypertensive medication. Each study participant received losartan 50 mg, bisoprolol 5 mg, hydrochlorothiazide 25 mg, and amlodipine 5 mg daily, each as a monotherapy in randomized order for 4 weeks. These drug treatment periods were preceded and separated from each other by 4 week placebo periods. Twenty four hour ambulatory blood pressure (ABP) readings were recorded at the end of each treatment period with a device equipped with a QRS complex detector and a position sensor (Diasys Integra; Novacor, Rueil Malmaison, France); in addition, office blood pressure measurements were carried out with repeated measurements after a 30 minute rest in the sitting position using a semiautomatic oscillometric device. The performance and quality control of ABP recordings have been described in detail earlier [23]. In short, readings were taken every 15 (standing) or every 30 min (recumbent). Single observations were excluded due to low pulse pressure, high heart rates, lying down during daytime, standing or being awake during night-time, or high physical activity [23]. A total of ≥15 daytime and ≥ 7 night-time readings were required for a recording to be accepted. The present study utilizes ABP response data for the 4 different monotherapies. The study was completed in altogether 228 men.

Blood plasma samples (collected in the morning under non-fasting conditions and stored at -80°C without any incidental thawings) for metabolite assays were available from 44 subjects in the following way: amlodipine, 38 samples; bisoprolol, 41 samples; hydrochlorothiazide, 39 samples; and losartan, 42 samples. The mean metabolite levels of 3 samples from the intervening placebo periods were used as baseline values in calculation of the drug effects; samples for only 2 placebo periods were available for one patient and for 1 placebo period for five patients. ABP recordings were evaluated in a fully blinded way as described earlier [23]; accepted recordings were available for 38 patients after placebo treatment and for 32–38 patients after each drug treatment.

The clinical part of the GENRES study was conducted in accordance with the Declaration of Helsinki and Guidelines for Good Clinical Practice (1996) at Helsinki University Central Hospital between years 1999 and 2004. The study was approved by the Ethical Committee of Helsinki University Central Hospital and the National Agency for Medicines of Finland. All subjects gave signed informed consent before any study related activities. The study is registered at ClinicalTrials.gov (NCT03276598).

### Identification and measurement of plasma metabolites

EDTA-anticoagulated plasma samples were stored at -80°C until processed. Sample processing was carried out as described previously [25] at Metabolon, Inc. Briefly, recovery standards were added prior to the first step in the extraction process for quality control purposes. Proteins were precipitated with methanol under vigorous shaking for 2 min (Glen Mills GenoGrinder 2000) followed by centrifugation. The resulting extract was divided into five fractions for analysis by ultra high performance liquid chromatography-tandem mass spectrometry (UPLC-MS/MS), as outlined below.

Three types of controls were analyzed in concert with the experimental samples: first, samples generated from a pool of human plasma extensively characterized by Metabolon, Inc. served as technical replicate throughout the data set; second, extracted water samples served as process blanks; and third, a cocktail of standards spiked into every analyzed sample allowed instrument performance monitoring. Instrument variability was determined by calculating the median relative standard deviation (RSD) for the standards that were added to each sample prior to injection into the mass spectrometers (median RSD = 5%; n = 31 standards). Overall process variability was determined by calculating the median RSD for all endogenous metabolites (i.e., non-instrument standards) present in 100% of the pooled human plasma matrix samples (median RSD = 9%; n = 485 metabolites).

Non-targeted MS (mass spectrometry) analysis was performed at Metabolon, Inc. Extracts were subjected to UPLC-MS/MS [25]. All methods utilized a Waters ACQUITY UPLC and a Thermo Scientific Q-Exactive high resolution/accurate mass spectrometer interfaced with a heated electrospray ionization source and Orbitrap mass analyzer operated at 35,000 mass resolution. The sample extract was vacuum dried then reconstituted in solvents compatible to each of the four methods. Each reconstitution solvent contained a series of standards at fixed concentrations to ensure injection and chromatographic consistency. One aliquot was analyzed using acidic positive ion conditions, chromatographically optimized for more hydrophilic compounds. In this method, the extract was gradient-eluted from a C18 column (Waters UPLC BEH C18-2.1x100 mm, 1.7 μm) using water and methanol, containing 0.05% perfluoropentanoic acid and 0.1% formic acid. The second aliquot was also analyzed using acidic positive ion conditions; however, it was chromatographically optimized for more hydrophobic compounds. In this method, the extract was gradient eluted from the same aforementioned C18 column using methanol, acetonitrile, water, 0.05% perfluoropentanoic acid and 0.01% formic acid and was operated at an overall higher organic content. The third aliquot was analyzed using basic negative ion-optimized conditions using a separate dedicated C18 column. The basic extracts were gradient eluted from the column using methanol and water, however with 6.5mM ammonium bicarbonate at pH 8. The fourth aliquot was analyzed via negative ionization following elution from a HILIC column (Waters UPLC BEH Amide 2.1x150 mm, 1.7 μm) using a gradient consisting of water and acetonitrile with 10mM ammonium formate, pH 10.8. The fifth aliquot was reserved for backup. The MS analysis alternated between MS and data-dependent MSn scans using dynamic exclusion. The scan range varied slightly between methods but covered 70–1000 m/z.

Metabolites were identified by automated comparison of the ion features in the experimental samples to a reference library of chemical standard entries that included retention time, molecular weight (m/z), preferred adducts, and in-source fragments as well as associated MS spectra and curated by visual inspection for quality control using software developed at Metabolon [26,27]. Identification of known chemical entities is based on comparison to metabolomic library entries of purified standards. Commercially available purified standard compounds have been acquired and registered into LIMS for distribution to the various UPLC-MS/MS platforms for determination of their detectable characteristics. Peaks were quantified using area-under-the-curve. Raw area counts for each metabolite in each sample were normalized to correct for variation resulting from instrument inter-day tuning differences by the median value for each run-day, therefore, setting the medians to 1.0 for each run. This preserved variation between samples but allowed metabolites of widely different raw peak areas to be compared on a similar graphical scale. Missing values were imputed with the observed minimum after normalization.

### Statistical analyses

Statistical analyses were done using IBM SPSS Statistics (v22). In these analyses, plasma metabolite levels were expressed as relative units: median of all analyzed samples was set to 1. In all analyses, the mean of three placebo periods was used as the metabolite baseline level.

The changes in metabolite concentrations caused by study drugs are expressed as percent change from baseline (mean of three placebo periods), and their statistical significance was analyzed using Wilcoxon signed-rank test.

Correlation between metabolite baseline levels and the antihypertensive effects of the four study drugs was analyzed using Pearson correlation. The residuals of normalized baseline metabolite levels (means of three placebo periods) were used here. They were calculated using stepwise regression with inclusion criterion of P < 0.10, and the tested covariates were age, body mass index, current smoking, and serum creatinine, serum glucose and daily urinary sodium excretion assessed after the first placebo period. Normalized baseline metabolite levels were used if there were no significant covariates. The antihypertensive responses of the study drugs were defined as the change in 24-hour ABP; means of all (up to four) placebo periods were used as the baseline levels. The effect of baseline covariates on the ABP responses was adjusted for by calculating residuals of systolic and diastolic ABP changes using stepwise regression with inclusion criterion of P < 0.10. The tested covariates were corresponding baseline ABP, age, current smoking, body mass index, daily urinary sodium excretion after the first placebo period, and serum creatinine, cholesterol, HDL cholesterol and triglyceride levels after the first placebo period. In addition to the corresponding (systolic or diastolic) baseline ABP level, the selected covariates were: for amlodipine systolic ABP response, age, daily urinary sodium excretion and serum cholesterol; for amlodipine diastolic ABP response, age, body mass index, daily urinary sodium excretion and serum cholesterol; for bisoprolol systolic ABP response, current smoking and serum cholesterol; for bisoprolol diastolic ABP response, no other covariates; for hydrochlorothiazide systolic ABP response, age, daily urinary sodium excretion and serum triglycerides; for hydrochlorothiazide diastolic ABP response, age and daily urinary sodium excretion; for losartan systolic and diastolic ABP responses, serum creatinine.

Correlation between the antihypertensive responses of the four drugs and changes in plasma metabolite levels was analyzed using partial correlation. Normalized value of the plasma metabolite change caused by the drug and the residual of ABP response (described above) were used. Normalized value of metabolite baseline level was used as the control variable.

Considering the >600 metabolites studied and concentration changes caused by 4 different drugs, a statistically significant change would require a P value of 2 x 10^−5^ (Bonferroni correction: 0.05/2400). However, we would consider even higher P values as potentially significant, provided there were metabolites which represent common pathways and/or belong to a similar biochemical family and which showed similar changes in response to a given drug. We used the same criteria in assessment of the correlation of blood pressure responses with placebo metabolite levels and changes in metabolite levels caused by the study drugs.

## Results

### General principles of data analysis

The dataset comprises 617 compounds of known identity that were measured in plasma samples of approximately 40 individuals after 1-month treatment of amlodipine (5 mg daily), bisoprolol (5 mg daily), hydrochlorothiazide (25 mg daily) and losartan (50 mg daily), each as a monotherapy in a rotational double-blind fashion, and after the preceding and intervening 1-month placebo periods (the average values of three placebo periods were used as the baseline reference). The baseline characteristics and antihypertensive drug responses of the study subjects are listed in Table 1.

**Table 1:** Characteristics of the study subjects.

Number of study subjects	44
Age, y	48.9 ± 6.7
Body mass index, kg/m^2^	27.3 ± 2.9
Fasting serum glucose*, mmol/L	5.1 ± 0.6
Serum creatinine*, μmol/L	91.4 ± 9.5
Urinary excretion of sodium*, mmol/24 h	160 ± 59
Blood pressure during placebo periods, mmHg	
Systolic ABP (n = 43)	134 ± 10
Diastolic ABP (n = 43)	92 ± 7
Systolic OBP (n = 44)	150 ± 12
Diastolic OBP (n = 44)	100 ± 7
ABP responses, mmHg	
Amlodipine, systolic (n = 37)	-5.9 ± 6.9
Amlodipine, diastolic (n = 37)	-4.4 ± 3.4
Bisoprolol, systolic (n = 38)	-10.2 ± 6.7
Bisoprolol, diastolic (n = 38)	-7.7 ± 4.6
Hydrochlorothiazide, systolic (n = 36)	-5.5 ± 6.0
Hydrochlorothiazide, diastolic (n = 36)	-2.2 ± 4.0
Losartan, systolic (n = 32)	-8.0 ± 5.9
Losartan, diastolic (n = 32)	-5.6 ± 3.7

Data are presented as mean±SD.

*Fasting serum glucose and creatinine, and daily urinary sodium excretion values are from clinical laboratory analyses after the first placebo period of the study. ABP, 24-hour ambulatory blood pressure; OBP, office blood pressure.

The test panel included amino acids and peptides (171 biochemicals), carbohydrates (20 biochemicals), energy/tricarboxylic acid cycle (TCA) metabolites (8 biochemicals), lipids (260 biochemicals), nucleotides (27 biochemicals), vitamins and cofactors (25 biochemicals) and xenobiotics (106 biochemicals) (S1 Table). Wilcoxon signed-rank test was used to identify changes in biochemical levels showing statistical significance; the results are listed in S1 Table. We carried out a detailed analysis to investigate first, individual drug effects on the different metabolite levels, second, the correlation of the average placebo levels of the metabolites to the blood pressure–lowering effects of the individual drugs, and third, the correlation between the drug-induced change (increase or decrease) of a given metabolite to the corresponding drug-induced change in blood pressure. In the latter two analyses, we used separately systolic and diastolic ambulatory blood pressure values.

### Effect of the four antihypertensive drugs on the levels of specific plasma metabolites

**Fig 1:**
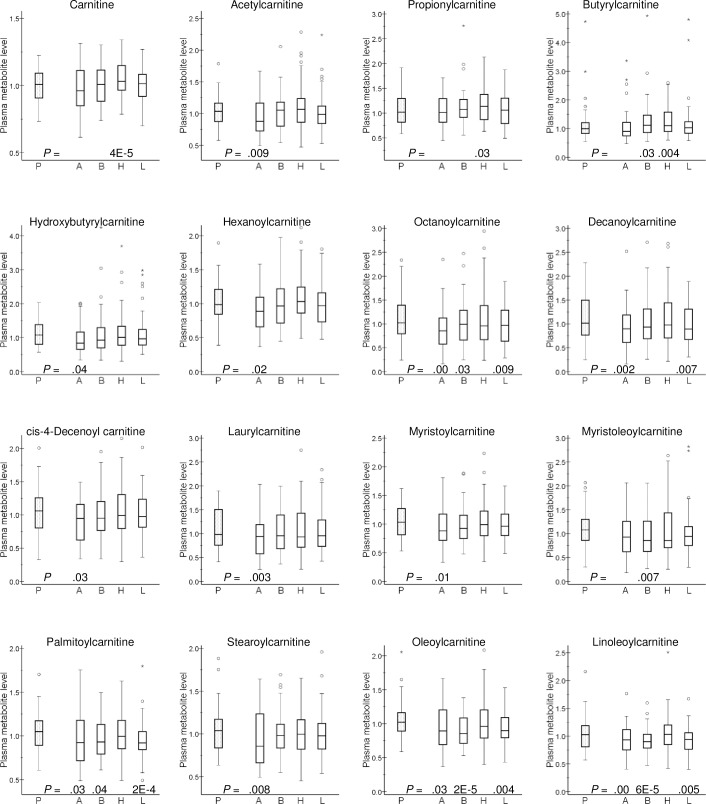
Effect of antihypertensive drugs on selected plasma acylcarnitines. Plasma metabolite level is presented as relative units: the median of all analyzed samples was set to 1. Box-and-whisker plots are presented. P values <0.05 from Wilcoxon signed-rank test are included. P, placebo (mean of three periods); A, amlodipine; B, bisoprolol; H, hydrochlorothiazide; L, losartan.

**Fig 2:**
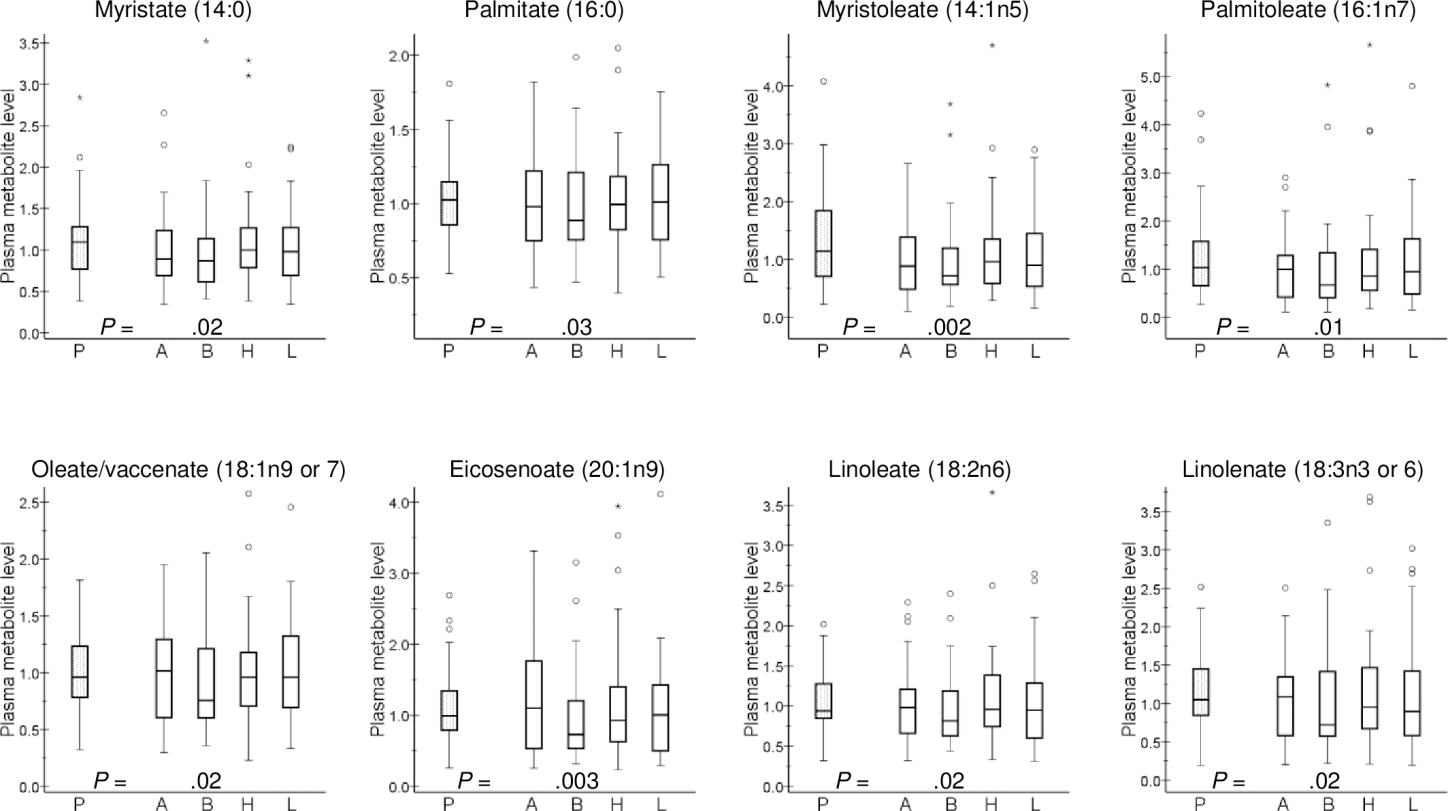
Effect of antihypertensive drugs on selected plasma long-chain fatty acids. Plasma metabolite level is presented as relative units: the median of all analyzed samples was set to 1. Box-and-whisker plots are presented. P values <0.05 from Wilcoxon signed-rank test are included. P, placebo (mean of three periods); A, amlodipine; B, bisoprolol; H, hydrochlorothiazide; L, losartan.

Amlodipine administration caused a notably consistent decrease of plasma levels of acylcarnitines (P values down to 0.001), without affecting the level of the parent compound (carnitine) itself (Fig 1). Likewise, bisoprolol therapy decreased the longer forms of acylcarnitines (P values down to 2 x 10^−5^), while this negative effect was less pronounced on acylcarnitines with shorter fatty acid chains (Fig 1). Furthermore, bisoprolol tended to decrease plasma levels of a number of long-chain fatty acids, including myristoleate, palmitoleate, oleate, eicosenoate and linoleate (with P values 0.003 to 0.02, Fig 2), as well as plasma phosphate (P = 6 x 10^−5^) (S1 Table).

**Fig 3:**
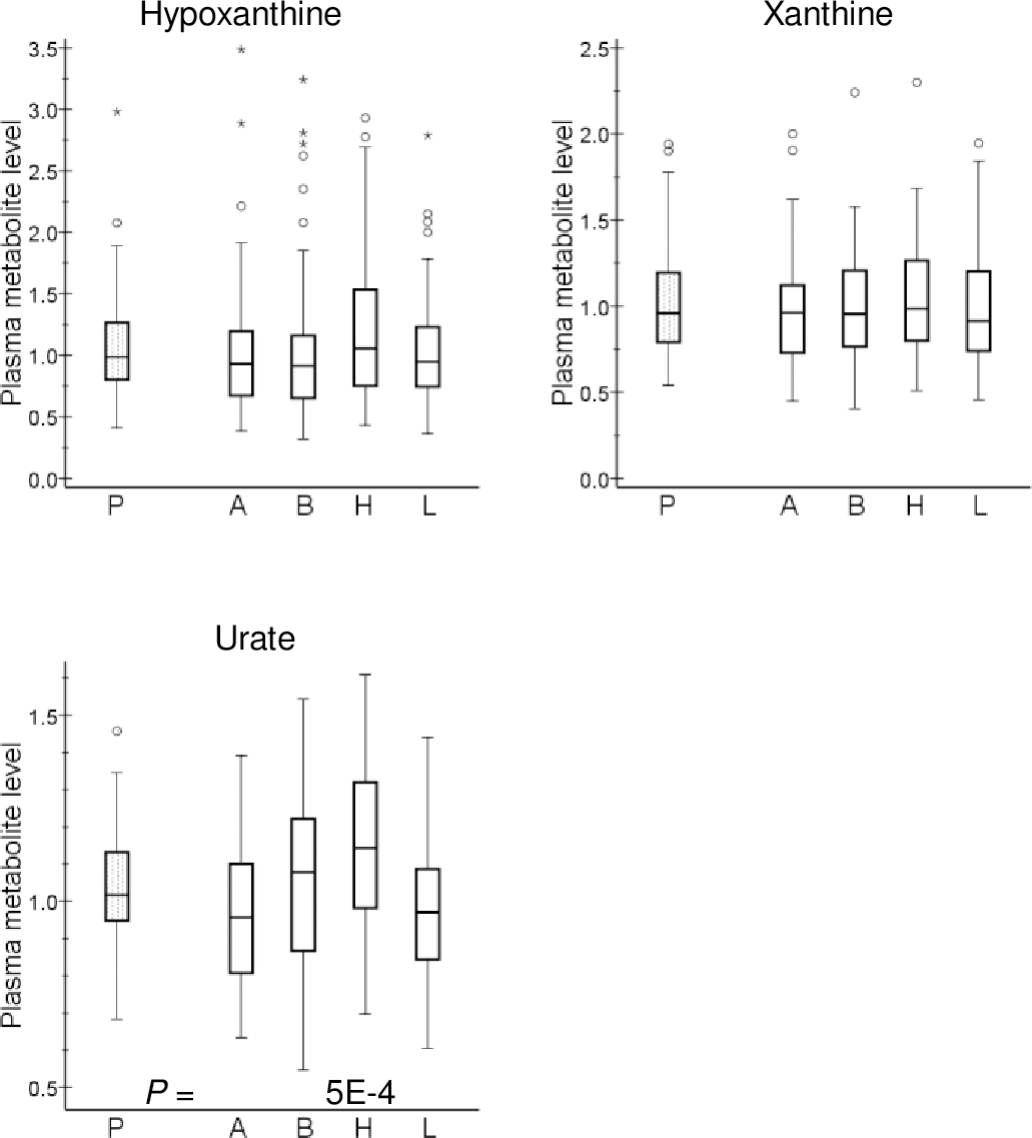
Effect of antihypertensive drugs on uric acid and its precursors. Plasma metabolite level is presented as relative units: the median of all analyzed samples was set to 1. Box-and-whisker plots are presented. P values <0.05 from Wilcoxon signed-rank test are included. P, placebo (mean of three periods); A, amlodipine; B, bisoprolol; H, hydrochlorothiazide; L, losartan.

In contrast, hydrochlorothiazide therapy did not result in marked alterations of plasma long-chain acylcarnitine levels, but slightly increased plasma carnitine (P = 4 x 10^−5^), propionylcarnitine (P = 0.03) and butyrylcarnitine (P = 0.004) levels (Fig 1). In addition, this thiazide diuretic tended to increase the urea cycle metabolites urea and citrulline but not ornithine and arginine (S1 Fig). Homocitrulline, a urea cycle-related amino acid, was increased by hydrochlorothiazide (P = 0.01) and decreased by amlodipine (P = 9 x 10^−4^) in the absence of significant effects on plasma creatinine levels (S1 Table). Furthermore, hydrochlorothiazide therapy brought about a relatively consistent increase of several androgenic steroid sulfates, which was paralleled by an increase in plasma inorganic sulfate level (S2 Fig). Not unexpectedly, thiazide therapy caused a 13% increase of plasma uric acid level (P = 5 x 10^−4^), without markedly affecting the levels of uric acid precursor compounds hypoxanthine and xanthine (Fig 3).

Similar to the effects of bisoprolol and amlodipine, losartan administration caused a decrease in the circulating levels of medium- and long-chain acylcarnitines (P values down to 2 x 10^−4^) (Fig 1).

### Baseline metabolite levels and antihypertensive responses of the four drugs

In search for possible predictive biomarkers for a favorable antihypertensive effect, we correlated the individual baseline metabolite levels (mean values of the placebo periods for a given subject) to the corresponding systolic and diastolic responses, analyzed separately for each of the four types of monotherapy. While we did not record correlations reaching P values < 2 x 10^−5^, we observed a trend for higher plasma levels of a number of aromatic amino acids (including phenylpyruvate, phenylalanine and N-acetylphenylalanine) to predict better antihypertensive responses to bisoprolol (with r values -0.48 to -0.31, and P values 0.002 to 0.06), and lower levels of plasma serotonin and phosphoethanolamine to predict a better systolic response to amlodipine (with r values 0.50 and 0.48, and P values 0.002 and 0.003, respectively). Calculations of correlations between baseline metabolite levels to hydrochlorothiazide or losartan responses mostly yielded less significant data.

### The correlation between the antihypertensive responses of the four drugs and changes in the plasma levels of the metabolites

**Fig 4:**
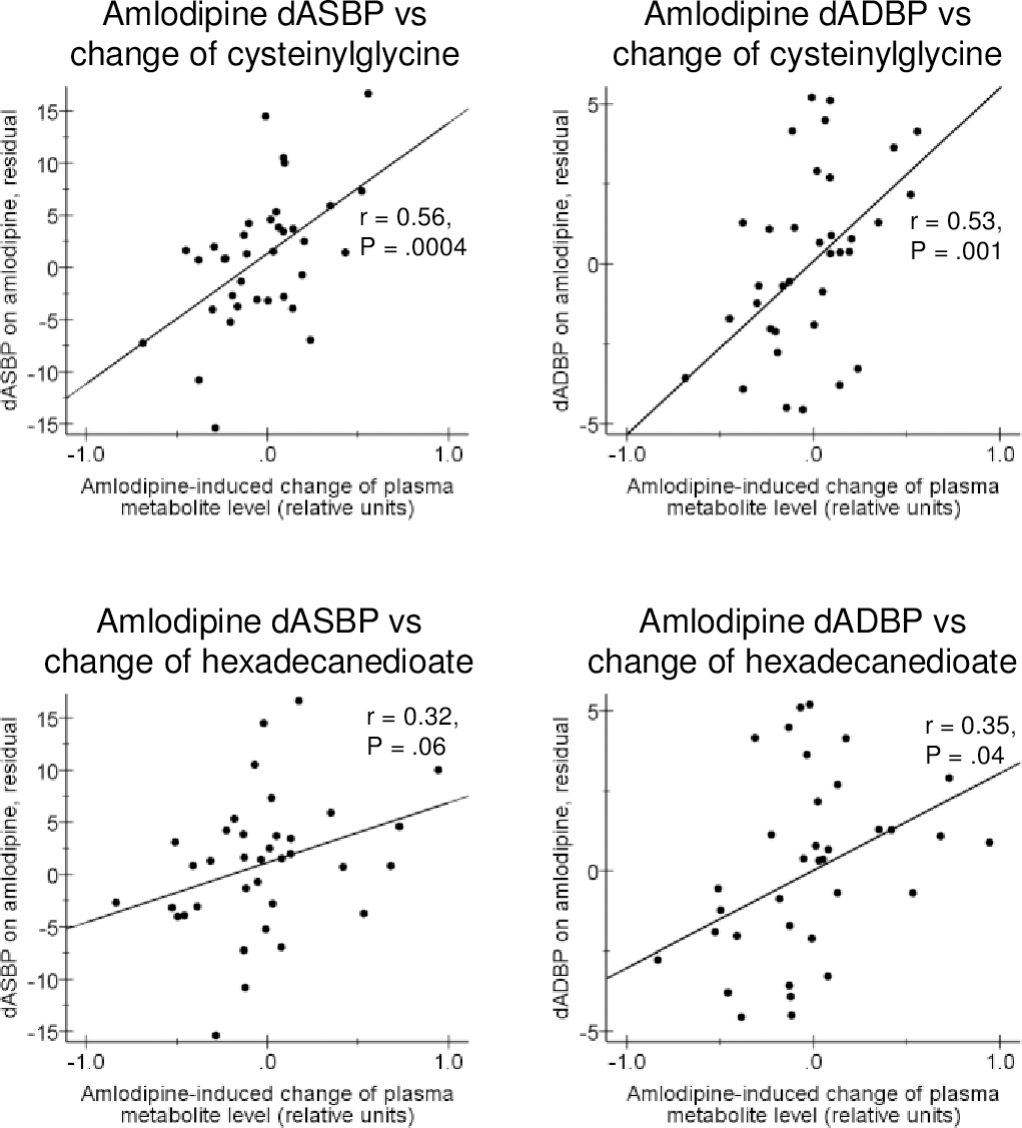
Correlation of the change of plasma cysteinylglycine and hexadecanedioate levels with the antihypertensive effect of amlodipine. Correlation coefficients (r) and P values from partial correlation, calculated with normalized metabolite change values and controlling for metabolite baseline level, are included. dASBP, change of 24-hour ambulatory systolic blood pressure; dADBP, change of 24-hour ambulatory diastolic blood pressure.

When amlodipine effects were studied and both systolic and diastolic blood pressure responses were noted, the most consistent effects were noted for two metabolites: decreases of systolic and diastolic blood pressure were associated with a decrease of plasma cysteinylglycine (P = 0.0004 and 0.001, respectively) and, to somewhat lesser extent, of hexadecanedioate (P = 0.06 and 0.04, respectively) levels (Fig 4). The relationships between other organic dicarboxylic acids and amlodipine effects were less marked.

In the case of bisoprolol, the most significant correlation emerged between systolic (P = 0.02) and diastolic (P = 0.005) blood pressure lowering vs. decrease in plasma fructose level (S3 Fig).

When a similar analysis of metabolite changes upon hydrochlorothiazide therapy was conducted, less significant correlations to both systolic and diastolic blood pressure responses were observed. On the other hand, calculations of relations between losartan-induced blood pressure responses and metabolite level changes revealed correlations between lowering of systolic and diastolic blood pressure vs. decrease in plasma oleamide (P = 0.004 and 0.02, respectively) and linoleamide (P = 0.004 and 0.03, respectively) (S4 Fig).

## Discussion

Even though numerous antihypertensive drugs are available for treatment of essential hypertension, many patients do not achieve proper control [3,4]. Currently, there is a lack of a set of predictive biomarkers to help determine which anti-hypertensive drug mechanism and which drug itself may be best suited for a given individual. This study examined plasma metabolites in individuals treated with four different anti-hypertensive drugs relative to placebo controls. The tested drugs were typical and widely used representatives of four major classes of blood pressure-lowering agents: hydrochlorothiazide (diuretic); amlodipine (Ca^+2^ channel blocker); bisoprolol (beta blocker) and losartan (angiotensin II receptor antagonist). The GENRES study has a cross-over design with subjects being treated with all four drugs, each as a monotherapy, and placebo wash-out periods occurred between treatments with the different drugs. Placebo comparisons were conducted using an averaged placebo value composed of three placebo plasmas collected from an individual subject.

In general, the magnitude of metabolite differences between drug-treated and placebo plasmas was relatively small. However, long- and medium-chain acylcarnitines were found to be lower in most drug-treatment groups relative to placebo periods, except for hydrochlorothiazide, which resulted in marginal decreases only (Fig 1). The homeostasis of L-carnitine and its esters is maintained in a careful and narrow fashion in the human body (for review, see [28]). Carnitine moieties are added to fatty acids to facilitate transport across the mitochondrial membrane for fatty acid β-oxidation. Decreases in long- and medium-chain acylcarnitines such as palmitoylcarnitine and hexanoylcarnitine can represent a decreased level of fatty acid β-oxidation, since acylcarnitine formation is a rate-limiting step in β-oxidation. Most measured long- and medium-chain acylcarnitines were lower in drug-treated relative to placebo plasmas for each of the drugs. Interestingly, the short-chain acylcarnitines butyrylcarnitine and propionylcarnitine tended to increase in all but the amlodipine plasmas, which runs counter to the directional change of the long- and medium-chain acylcarnitines. Increased circulating levels of acylcarnitines, particularly long-chain acylcarnitines, have been previously linked to increased risk to predict cardiovascular events in patients at risk for coronary heart disease [29–31]. The potential association of carnitine metabolism to development and treatment of hypertension appears to be complex. Mostly based on studies in laboratory animals, L-carnitine itself has been proposed as a protective agent against cardiac and renal remodeling in arterial hypertension [32]. However, both serum L-carnitine and long-chain acylcarnitine levels were reported to be positively correlated to ambulatory blood pressure [33], and increased plasma carnitine as well as short- and long-chain acylcarnitine levels were measured in in patients with preeclampsia [34]. Increased long-chain acylcarnitine levels have also been associated with insulin resistance with diabetes, possibly via interference of insulin signaling within the cell membrane, while this relation is less clear for short-chain acylcarnitines [35]. Provided it is especially the long-chain acylcarnitines that may be associated with adverse cardiometabolic effects, it is of some interest that in our study the thiazide diuretic, known to induce insulin resistance, did not markedly reduce plasma long-chain acylcarnitines.

Short-chain acylcarnitines can also be generated from branched-chain amino acid metabolism and may not be completely reflective of generation from fatty acid β-oxidation. If increased fatty acid β-oxidation or decreased TCA cycle activity leads to an excess of acetyl-CoA, ketogenesis can occur resulting in the formation of ketone bodies such as 3-hydroxybutyrate (BHBA). Similarly, a reduction in acetyl-CoA concentration may lower ketogenesis and BHBA levels. BHBA levels were 17% lower in plasma samples after bisoprolol treatment compared with averaged placebos (P = 0.007) (S1 Table).

A group of sulfated steroids were elevated after hydrochlorothiazide administration (S2 Fig). The significance of this finding remains obscure, as sulfated steroids as such are proposed to act as a steroid reservoir in blood plasma, with no ability as hydrophilic molecules to penetrate target cell membrane and to interact with steroid receptors [36,37]. The most abundant sulfated steroid dehydroepiandrosterone sulfate may have impact on blood pressure regulation, but its levels were not significantly affected by the four drugs tested in the present study (S2 Fig).

Hydrochlorothiazide and amlodipine induced interesting and partially diverse effects on urea cycle-related metabolites (S1 Fig). Citrulline, an intermediate of urea biosynthesis, and urea were elevated upon hydrochlorothiazide administration (S1 Fig, S1 Table). Similar findings were published as early as in 1969 for urea [38]. There appears to be no such data for citrulline. Urea cycle function is of interest regarding hypertension because of the production of the vasoactive compound nitric oxide from arginine and homoarginine. However, these two precursor amino acids were not altered by thiazide therapy. Homocitrulline (ε-carbamoyl lysine) may be generated *in vivo* through a pathway involving decomposition of urea to ammoniac and cyanate. Cyanate in turn is rapidly converted to a highly reactive isocyanic acid, which then reacts with the ε-amino group of lysine, forming homocitrulline. It may also be produced at sites of inflammation through the action of myeloperoxidase [39]. Therefore, homocitrulline may serve as a marker of protein carbamoylation, which may affect the function of proteins and mediate, e.g. some of the deleterious effects of uremia [40]. Elevated plasma protein-bound homocitrulline concentrations predict increased risk of coronary artery disease, future myocardial infarction, stroke and death [39], and elevated free serum homocitrulline concentration is associated with poor coronary collateral growth in coronary heart disease patients [41]. However, it remains unclear whether reduction of homocitrulline levels by amlodipine (median change -21%, P = 9 x 10^−4^) and elevation of its levels by hydrochlorothiazide (median change +16%, P = 0.004) have any relation to risk reduction of cardiovascular events during antihypertensive treatment.

The search for possible predictive baseline metabolic markers for antihypertensive drug responsiveness turned out to result in somewhat disappointing data. No metabolite in placebo plasma samples was shown to associate with an antihypertensive drug response at statistical significance level of P < 2 x 10^−5^. The potential association of baseline levels of aromatic amino acids, phenylalanine and N-acetylphenylalanine in particular, as already reported by us [42], to a favorable response to bisoprolol is of some interest due to our previous findings of association between *ACY3* (coding for aminoacylase 3) variation and bisoprolol responsiveness [13]; aminoacylase 3 is the enzyme catalyzing conversion of N-acetylphenylalanine to phenylalanine. It is of note that while the levels of a number of long-chain acylcarnitines were diminished in a relatively systematic fashion by amlodipine, bisoprolol and losartan, their baseline levels were not predictive for a good antihypertensive response to any of these drugs.

The fact that both systolic and diastolic blood pressure responses to amlodipine treatment were correlated to decreases in plasma hexadecanedioate concentration (Fig 4) is intriguing due to recent data by Menni et al. [21], linking this metabolite to blood pressure regulation. Indeed, circulating hexadecanedioate showed association with systolic and diastolic blood pressure levels in three different patient cohorts (TwinsUK, KORA and Hertfordshire, altogether appr. 8 000 individuals) and was also associated with mortality in TwinsUK sample. Furthermore, administration of hexadecanedioate to Wistar-Kyoto rats was demonstrated to increase blood hexadecanedioate as well as blood pressure. These authors also showed that this metabolite increased vascular reactivity to noradrenaline in these animals [21]. Considering the marked changes of plasma acylcarnitines induced by amlodipine in the present study (Fig 1), it is of interest that Menni et al. [21] also observed an inverse correlation between plasma hexadecanedioate and carnitine levels. More recently, elevated levels of hexadecanedioate were associated with increased blood pressure levels and risk of incident heart failure in both African-Americans and an independent sample of European Americans [22]. Collectively, all these data tend to link fatty acid transport and oxidation mechanisms to blood pressure regulation and, possibly, to the antihypertensive action of some antihypertensive drugs including amlodipine.

There are only scant previous systematic studies on blood pharmacometabolomics in human hypertension. The PEAR investigators reported certain racial differences in blood metabolite profile following antihypertensive drug administration and found that the levels of a number of organic acids, including oleic, linoleic, palmitoleic, palmitic, BHBA and myristic acid, were significantly decreased upon atenolol treatment of white and, to slightly lesser extent, of black patients [18]. Interestingly, we could replicate all of the latter findings in our study using bisoprolol as our beta blocker (Fig 2), thus further supporting the idea that fatty acid metabolism may have important role in blood pressure control by antihypertensive agents. Due to marked differences in the metabolite panel assayed by us and that by the PEAR Study group [19,20], it is not possible to systematically compare the metabolite signatures after thiazide treatment in these two studies. However, we found a general increase of the urea cycle metabolites after hydrochlorothiazide administration, which appears to be in line with the data of the PEAR group [19]. In contrast to the data of Rotroff et al. [19], we did not see marked differences in plasma levels palmitoleic and arachidonic acid upon thiazide treatment.

There are important limitations of the study. First of all, metabolite analyses were conducted in only 44 individuals, which may increase the probability of chance findings. It should be emphasized, however, that variation in the baseline levels could be significantly diminished by using the means of three placebo values. Even if a replication study following the exact design of the GENRES Study would be heavy to be conducted, we emphasize the need for replication reports coming e.g. from data bases of previously conducted studies where data on specific antihypertensive drugs and specific metabolites would be available. Second, the subjects were not asked to fast during sample collection, which may increase metabolite variability in plasma. We chose this policy intentionally, in order to avoid unavoidable and undesirable stress which could have affected the ongoing ABP and office BP measurements. Third, the GENRES Study was designed to involve male hypertensive patients only. The strengths of our study include its rotational design as well as the wide spectrum of biochemicals analyzed, which both should facilitate detection of overall differences in metabolic signatures caused by different classes of antihypertensive agents.

In conclusion, treatment of male subjects with four antihypertensive medications, each with different mechanisms of action, resulted in subtle changes to their plasma metabolite profiles, relative to placebo controls. The most generalized metabolite response to anti-hypertensive treatment was a decline in the levels of acylcarnitines, in particular by a calcium channel and a beta blocker, indicative of a change in fatty acid metabolism. Another finding of special note was the association of the antihypertensive effect of amlodipine to the decline in plasma hexadecanedioate, a metabolite recently strongly associated with blood pressure regulation. Overall, our data indicate that treatment with anti-hypertensive agents result in limited changes to plasma metabolite profiles. Some metabolite changes more prominent to specific drugs may offer some insights into how different mechanisms of action impact plasma metabolite homeostasis.

## Supporting information

S1 Table Drug-induced changes in biochemical concentrations. (XLSX)

S1 Fig Effect of antihypertensive drugs on urea cycle metabolites. (DOCX)

S2 Fig Effect of antihypertensive drugs on selected plasma androgenic steroid sulfates and inorganic sulfate. (DOCX)

S3 Fig Correlation of the change of plasma fructose level with the antihypertensive effect of bisoprolol. (DOCX)

S4 Fig Correlation of the change of plasma oleamide and linoleamide levels with the antihypertensive effect of losartan. (DOCX)
