# Supplementary material for: Effects of four different antihypertensive drugs on plasma metabolomic profiles in patients with essential hypertension
Source: PLoS One. 2017 Nov 9;12(11):e0187729. doi: 10.1371/journal.pone.0187729 (PMC5679533; doi:10.1371/journal.pone.0187729)
Supplement: S2 Fig — (DOCX) [file pone.0187729.s003.docx]

**S2 Fig.** **Effect of antihypertensive drugs on selected plasma androgenic steroid sulfates and inorganic sulfate.**

Data for metabolites with *P* values <0.001 in response to hydrochlorothiazide treatment and dehydroepiandrosterone sulfate are shown. Plasma metabolite level is presented as relative units: the median of all analyzed samples was set to 1. Box-and-whisker plots are presented. *P* values <0.05 from Wilcoxon signed-rank test are included. P, placebo (mean of three periods); A, amlodipine; B, bisoprolol; H, hydrochlorothiazide; L, losartan.
