# Supplementary material for: Effects of four different antihypertensive drugs on plasma metabolomic profiles in patients with essential hypertension
Source: PLoS One. 2017 Nov 9;12(11):e0187729. doi: 10.1371/journal.pone.0187729 (PMC5679533; doi:10.1371/journal.pone.0187729)
Supplement: S3 Fig — (DOCX) [file pone.0187729.s004.docx]

**S3 Fig. Correlation of the change of plasma fructose level with the antihypertensive effect of bisoprolol.**

Correlation coefficients (*r*) and *P* values from partial correlation, calculated with normalized metabolite change values and controlling for metabolite baseline level, are included. dASBP, change of ambulatory systolic blood pressure; dADBP, change of ambulatory diastolic blood pressure.
